# Factors affecting study efficiency and item non-response in health surveys in developing countries: the Jamaica national healthy lifestyle survey

**DOI:** 10.1186/1471-2288-7-13

**Published:** 2007-02-28

**Authors:** Rainford Wilks, Novie Younger, Jasneth Mullings, Namvar Zohoori, Peter Figueroa, Marshall Tulloch-Reid, Trevor Ferguson, Christine Walters, Franklyn Bennett, Terrence Forrester, Elizabeth Ward, Deanna Ashley

**Affiliations:** 1 Tropical Medicine Research Institute, the University of the West Indies, Mona, Kingston 7, Jamaica; 2 The Ministry of Health, Kingston Mall, Kingston, Jamaica

## Abstract

**Background:**

Health surveys provide important information on the burden and secular trends of risk factors and disease. Several factors including survey and item non-response can affect data quality. There are few reports on efficiency, validity and the impact of item non-response, from developing countries. This report examines factors associated with item non-response and study efficiency in a national health survey in a developing Caribbean island.

**Methods:**

A national sample of participants aged 15–74 years was selected in a multi-stage sampling design accounting for 4 health regions and 14 parishes using enumeration districts as primary sampling units. Means and proportions of the variables of interest were compared between various categories. Non-response was defined as failure to provide an analyzable response. Linear and logistic regression models accounting for sample design and post-stratification weighting were used to identify independent correlates of recruitment efficiency and item non-response.

**Results:**

We recruited 2012 15–74 year-olds (66.2% females) at a response rate of 87.6% with significant variation between regions (80.9% to 97.6%; p < 0.0001). Females outnumbered males in all parishes. The majority of subjects were recruited in a single visit, 39.1% required multiple visits varying significantly by region (27.0% to 49.8% [p < 0.0001]). Average interview time was 44.3 minutes with no variation between health regions, urban-rural residence, educational level, gender and SES; but increased significantly with older age category from 42.9 minutes in the youngest to 46.0 minutes in the oldest age category. Between 15.8% and 26.8% of persons did not provide responses for the number of sexual partners in the last year. Women and urban residents provided less data than their counterparts. Highest item non-response related to income at 30% with no gender difference but independently related to educational level, employment status, age group and health region. Characteristics of non-responders vary with types of questions.

**Conclusion:**

Informative health surveys are possible in developing countries. While survey response rates may be satisfactory, item non-response was high in respect of income and sexual practice. In contrast to developed countries, non-response to questions on income is higher and has different correlates. These findings can inform future surveys.

## Background

Jamaica has a population of approximately 2.6 million and has undergone a significant demographic transition in the last 5 decades [1-3]. Some features of this transition include the increase in the median age of the population from 17 years to 25 years between 1970 and 2000, the doubling of the proportion of persons older than 60 years old to over 10% and the increase in life expectancy at birth from less than 50 years in 1950 to greater than 70 years in 2000 [4]. As a result, the main causes of illness and death in Jamaica and many other Caribbean islands and regions at a similar state of development are the chronic non-communicable diseases (CNCDs)[3,5-8].

Most of the evidence to document this transition has come from routine sources of data. For instance the Statistical Institute of Jamaica using data obtained from the Registrar Generals Departments reports that cardiovascular diseases, cancer, diabetes mellitus, injuries and HIV related diseases are among the leading causes of death [4]. Data on morbidity is less readily available but analysis of hospital discharges confirm that a similar range of diseases make up the leading causes of hospitalization [9]. Hospital discharges and mortality data do not provide timely, reliable or complete information on disease burden and in countries like Jamaica routine morbidity statistics are not readily available. Thus periodic surveys are required to provide data on secular trends and provide information for policy development and assessment of the impact of interventions[10].

Health surveys are faced with several challenges, which include the ability to obtain representative samples, the accuracy of instruments, the reliability of observers and the willingness of respondents to participate and to provide valid responses [11,12]. Data on socio-economic status, family medical history and sexual behaviour facilitate quantification of health risk and inform policy development in communities. The validity of these data, which are required for planning culturally appropriate interventions, is often compromised by non-participation of selected subjects and non-response to certain questionnaire items. There are few reports on factors associated with survey and item non-response in developing countries. In previous reports from developed countries income non-response has been recognized as a potential source of bias and may vary with age and labour force status [13]. Questions on sexual practice are also subject to high non-response [14] but there are no data from our region. In this report we examine the experiences from a national health survey carried out between July 2000 and June 2001 in Jamaica, a middle-income developing country. We report on the logistical issues which affect health surveys and item non-response which may affect the validity of the data.

## Methods

### Sample, sampling methods and setting

We aimed to select a nationally representative sample of persons 15–74 years old, while having sufficient numbers to detect trends and generate hypotheses at the parish and regional levels. Based on previous prevalence estimates of 25% and 12% for hypertension and diabetes respectively, 2000 respondents would be adequate to derive a national estimate of the prevalence of these conditions with a 2% margin of error with a 95% confidence interval [15,16].

Jamaica is divided into 14 parishes and the Ministry of Health (MoH) has divided the country into 4 Regional Administrative Health Authorities (RHAs); viz. South East (SERHA), South (SRHA), North East (NERHA) and West (WRHA). Enumeration districts (ED's), consisting of up to 400 households, are used as primary sampling units (PSUs) as in other studies [4]. In previous surveys 36 persons were selected per ED and thus 56 ED's would yield a sample of 2016 subjects [17]. Using the STATIN Labour Force Sample of 478 EDs a sampling fraction of 56/478 was used to select EDs in each of the four regions and in each parish in a multistage sampling design. Using this fixed proportion in each parish resulted in larger parishes having greater number of ED's. Within each parish, a systematic random sampling method was used to select individual ED's (PSUs). Thirty-six persons were selected per ED and thus 56 ED's would yield a sample of 2016 subjects [17].

Within each ED the recruitment would begin at a designated point selected by STATIN and interviewers would proceed from house to house in a clockwise manner in circles of decreasing radii until the sample is achieved or the ED is exhausted. Kish Random Selection Method of sub-sampling [18] was used to identify a single subject from each household thereby facilitating independence of responses. If for any reason the selected household member could not participate or declined to participate, no other member of the household could replace that person. In the case where the selected person was unavailable or not at home, a minimum of three call-back visits would be made to the household at different times of day before moving to the next household to seek a replacement. Persons were considered resident in the household if they slept at the house at least three nights per week.

### Project Administration

The project was administered through a collaborative effort of the Ministry of Health (Jamaica) and the Epidemiology Research Unit of the Tropical Medicine Research Institute (TMRI), University of the West Indies. The team, consisting of statisticians, epidemiologists and nutritionists, had responsibility for survey design, instrument development and project oversight and data management. A national coordinator had direct responsibility for field operations.

Prior to the start of the study the team sought to increase public awareness about the study through radio interviews, press releases in the print media, the distribution of flyers in selected EDs and word-of-mouth promotion by influential persons in the communities. In response to tension resulting from violent episodes unrelated to the survey, community leaders aided safe entry of the SERHA field team into some communities in Kingston and St. Andrew. In some areas in St. Catherine Community Health Aides (CHAs) assisted with recruitment thereby improving response rates. Both CHAs and community leaders were trained in the per-protocol recruitment procedures. Care was taken to prevent these innovations from interfering with the selection of households and individuals or from compromising informed consent.

A regional team supervisor directed interview teams in each health region through monthly meetings and weekly telephone contact during the course of data collection. Weekly data logs tracked the progress of data collection. The South East region was assigned two team supervisors because of its larger sample size.

### Questionnaire Reliability, Training, and Certification

Test-retest reliability of the questionnaire was conducted prior to the main study. Twenty (20) respondents who were not participants in the main study were interviewed on two occasions seven days apart. Reliabilities were determined by percent agreement.

Members of the RHA health care staff (mainly nurses, nutritionists and dieticians), were employed to the study as field workers. All field workers completed a three day training programme, in which they were instructed and certified according to standard protocols in the measurement of blood pressure, anthropometry, questionnaire administration and capillary blood sampling for fasting glucose and total cholesterol estimation. Quality control was established by ensuring reliability between observers.

### Measurements

On the initial home visit after the selected eligible participant gave informed consent, questionnaire administration gathered demographic data, family, medical and social history as well as data on lifestyle practices, physical activities and dietary habits. Blood pressure was measured from the right arm of the seated subject after five minutes rest and was recorded to the nearest 2 mmHg using 1^st ^and 5^th ^Korotkoff sounds. Anthropometric measurements were also made including height, and waist and hip circumferences and were recorded to the nearest 0.1 cm and weight was recorded to the nearest 0.1 kg with the participants wearing light clothing and using instruments that were calibrated weekly. If measurement of subjects in their homes proved inconvenient, the subjects were measured at more suitable nearby settings, such as a church hall, school, community or health centre. On a follow-up visit the subject provided finger-prick blood samples for estimation of fasting blood glucose and total cholesterol using portable glucose and cholesterol measuring machines (GCT Accutrend).

### Data Analysis

**Figure 1:**
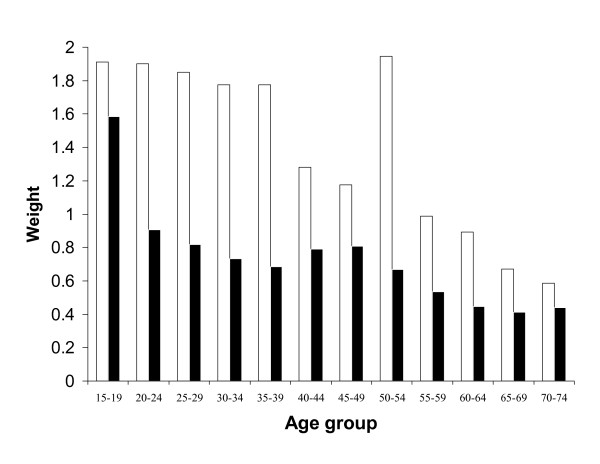
**Sample Weights By Age and Gender**. □ – Males – ■ Female.

The data were double entered using Access database, verified and cleaned. Data were analyzed using Stata version 8 [19]. Using gender specific 5-year age bands the distribution of the sample was adjusted to reflect the distribution of the national population using post-stratification weights (Fig. 1). Both weighted and un-weighted estimates of proportions and means are presented. Along with use of post-stratification weights, data analysis also took account of the multistage sampling design (four health regions and fourteen parishes), using the procedures available in Stata 8.

Univariate comparisons were done using the chi-squared statistic or Fisher's exact test where indicated and multivariable linear and logistic regression models were developed to explore potential explanatory variables (e.g. age and social status etc) for outcomes including non-response to questions on income and duration of interview. For this report, non-response to individual questions was defined as "no response" or a "don't know" as well as missing responses to the question. We also examined the profile of persons responding "don't know" compared to other non-responders.

Social status was subjectively assessed by interviewers and rated on a scale of 1–10. The responses were collapsed into three categories with ratings 1–3 as low social class, ratings 4–7 as middle social class and ratings 8–10 as high social class. Information on highest level of education was placed in three categories: basic school or lower; primary or all-age; and secondary or tertiary.

### Ethics

University of the West Indies, University Hospital of the West Indies and Ministry of Health Ethics Committees reviewed and approved the study. All participants gave written informed consent to all procedures prior to participation.

## Results

### Recruitment

We recruited 2012 individuals, ages 15–74 years, over a twelve month period (2000–2001). There was very little difference between the projected and actual number of EDs selected for recruitment. Three parishes, two mainly rural, (Portland and St. Elizabeth with 77% and 86% rural dwellers respectively)[4], and St. Catherine (26% rural), each required an additional ED in order for the required sample to be obtained and this resulted in the survey taking place in 31 instead of 28 rural EDs. In Portland (77% rural) three rural EDs were studied instead of one urban and two rural ones. St. Andrew had twelve instead of eleven urban EDs. Except for St. Catherine which had the largest shortfall in recruited participants (11/396; 3%), recruitment was consistent with projections in all parishes (Table 1). Within and across parishes more females (66.2%) were in the sample with proportions ranging from 71.6% in Kingston to 57.3% in St. Mary. Regional differences in response rates (Table 1) were statistically significant (p < 0.0001) with the predominantly urban SERHA (78.4% urban residents) showing the lowest response rate and the predominantly rural NERHA (24.1% urban residents) the highest (Table 1).

**Table 1:** Sampling projections and final recruitment by region and parish

**Health Region**	**Parish**	**Projected Sample**	**Actual Number Recruited**			**Recruitment effort**	
			M	F	Total	#Contacted	**Resp. rate **
		***(n)***	*n (%)*	*n (%)*			(%)

	Kingston	36	10 (28.6)	25(71.4)	35		
	St. Andrew	432	124(28.4)	312(71.6)	436		
	St. Thomas	72	23(31.9)	49(68.1)	72		
	St. Catherine	396	128(33.2)	257(66.8)	385		
SERHA		**936**			**928**	1147	80.9
	Portland	108	44 (40.7)	64 (59.3)	108		
	St. Mary	72	32 (42.7)	43 (57.3)	75		
	St. Ann	144	54 (37.5)	90 (62.5)	144		
NERHA		**324**			**326**	334	97.6
	Trelawny	36	15 (40.5)	22 (59.5)	38		
	St. James	144	54 (37.5)	90 (62.5)	144		
	Hanover	108	26 (24.1)	82 (75.9)	108		
	Westmoreland	108	34 (31.5)	74 (68.5)	108		
WRHA		**396**			**398**	413	96.4
	St. Elizabeth	72	24 (33.3)	48 (66.7)	72		
	Manchester	108	38 (35.2)	70 (64.8)	108		
	Clarendon	180	74 (41.1)	106 (58.9)	180		
SRHA		**360**			**360**	402	89.5
**TOTAL**		**2016**	**680 (33.8)**	**1332(66.2)**	**2012**	**2296**	**87.6**

SERHA = South East Regional Health Authority;

NERHA = North East Regional Health Authority

WRHA = Western Regional Health Authority

SRHA = Regional Health Authority

Resp. Rate = Response Rate

The sample as a proportion of the population by region ranges from 0.10 to 0.14 per cent with an overall proportion of 0.12%, reflecting a consistent sample proportion in all regions (data not shown). The gender distribution in the sample differs significantly from the approximately 1:1 ratio in the Jamaican population. There are also discrepancies in age distribution; for example, while 15–34 year olds account for 43.4% of the sample, this age group accounts for 52.0% of the 15–74 year-olds in the population and persons aged 55 years and older account for 24.8% of the sample compared to 15.1% of the population. Application of derived weights to the sample yielded the age-gender distribution which is very similar to that of the Jamaica population (Table 2).

**Table 2:** Age and Sex Distribution of sample (Actual & Weighted*) and the Jamaican Population

	**Sample**						**Jamaica Population**		
	**Males**		**Females**		**Total**		**Males**	**Females**	**Total**

	**Unwtd**	**Wtd**	**Unwtd**	**Wtd**	**Unwtd**	**Wtd**			

	***n(%)***	***%***	***n(%)***	***%***	***n(%)***	***%***	***n(%)***	***n(%)***	***n(%)***

15–24	142(21.0)	28.7	249(18.8)	27.5	391(19.5)	28.1	231449(28.3)	236408(27.5)	467857(27.9)
25–34	140(20.7)	26.1	338(25.5)	25.8	478(23.9)	25.9	193240(23.6)	211237(24.6)	404477(24.1)
35–44	126(18.6)	20.0	273(20.6)	19.6	399(19.9)	19.8	163930(20.0)	176453(20.6)	340383(20.3)
45–54	85(12.6)	11.1	153(11.5)	12.4	238(11.9)	11.8	105526(12.9)	105414(12.3)	210940(12.6)
55–64	89(13.1)	7.7	159(12.0)	8.5	248(12.4)	8.1	70473(8.6)	70455(8.2)	140928(8.4)
65–74	95(14.0)	6.4	153(11.5)	6.1	248(12.4)	6.3	53757(6.6)	58214(6.8)	111971(6.7)
**Total**	**677**		**1325**		**2002**		**818375**	**858181**	**1676556**

Unwtd = Unweighted; Wtd = Weighted

* Weighted proportions were derived using post-stratification weights based on 5-year age-gender categories.

### Efficiency of Recruitment & Study Execution

The modal and median number of visits required to complete recruitment was one (1) in all regions but the non-parametric Kruskall-Wallis test gave evidence of a statistically significant regional differences in the distribution of the number of required visits. This statistical significance is a reflection of the differences in the distributions of the number of visits within the 75^th ^to the 99^th ^percentile (Table 3). Poisson regression analysis that accounted for sampling weights and design did not confirm these differences. There were regional differences in the proportion of subjects requiring multiple visits to secure recruitment, with the NERHA showing 49.8% of recruits requiring multiple visits compared to the SRHA where the proportion was 27.0% (p < 0.0001) (Table 3). However, these differences did not persist when weighted logistic regression was used.

**Table 3:** Summary statistics illustrating the efficiency of the enrolment (number of visits and duration of interview) in Regional Health Authorities (RHAs)

RHA	**# of Visits^1^**	**% requiring >1 visit**	**Mean Interview Time**
**South East**	2 – 4	37.3**	44.7(0.54)***
**North East**	2 – 4	49.8	54.2(1.39)
**South**	2 – 3	27.0	39.9(0.41)
**West**	2 – 5	45.8	42.6(0.68)
**Total**	**2 – 4**	**39.1**	**44.7(0.37)**

**p < 0.01 ***p < 0.001

^1^75^th ^to 99^th ^percentile

The unweighted mean time taken to complete the interviews was 44.7 minutes (95% CI: 43.9, 45.4) and there were significant differences between the health regions ranging from 39.9 minutes in the SRHA to 54.2 minutes in the NERHA (p < 0.001). The weighted analysis accounting for sampling design (data not shown) revealed no significant differences between regions and an overall mean duration of interview of 44.3 minutes (95% CI: 41.2, 47.4). There were significant differences across age groups with the youngest age group (15–24 y.o.) averaging 42.9 minutes compared to the oldest age groups (55–64 and 65–74 y.o.) 46.1 and 46.0 minutes respectively and the statistically significant difference persisted in the weighted analysis. There was a statistically significant linear trend across these age groups (p < 0.01). Urban residents appeared to require shorter time to complete interviews compared to rural residents (43.4 vs 45.9 minutes; p < 0.01) in unweighted analysis but this was not confirmed in the weighted analysis (43.0 vs 45.5 minutes).

In weighted multivariate analysis, accounting for sampling design with the predictors age (as a continuous variable), regional health authority, gender, employment status and perceived economic status, only age remained significantly associated with interview time (Table 4). Each additional year of age was associated with an additional 0.06 minutes (3.6 seconds, 95% CI: 0.8, 6.8 seconds) thus older persons can be expected to require longer time for interview administration. Age was shown to be negatively correlated with educational level (Spearman's correlation coefficient -0.46, p < 0.001) suggesting that younger persons achieved higher education. When education level was included in the above model age was no longer significantly associated with interview time. This was not corroborated by a significant relationship between education level and interview time.

**Table 4:** Increments in interview time (in minutes) associated with the variation in the respective explanatory variables.

	**Increment in Interview time**	
**Explanatory variable**	**Weighted estimates**	Un-weighted estimates

Age*	**0.06 (0.013, 0.11)**	0.05 (0.01, 0.09)
**Employed**	**1.08 (-0.61, 2.77)**	0.5 (-1.07, 2.11)
**Female**	**1.2 (-0.14, 2.61)**	0.8 (-0.76, 2.27)
**Socioeconomic Status**		
**Middle**	**0.3 (-2.50, 3.04)**	0.4 (-1.13, 1.90)
**High**	**8.2 (-1.55, 17.94)**	9.0 (6.38, 11.60)
**Regional Health Authority**		
**North East**	**7.8 (-4.73, 20.32)**	7.4 (5.24, 9.59)
**South**	**-4.3 (-9.50, 0.87)**	-4.5 (-6.44, -2.65)
**West**	**-1.8 (-8.66, 5.07)**	-2.2 (-4.10, -0.29)

Coefficients for employed, female, socioeconomic status and the health regions are increments relative to the unemployed, males, low status, and South East health region, respectively.

The test-retest reliability of the questionnaire ranged from 70 – 88% (per cent agreement) except for questions on family history where reliability ranged from 25 – 33%.

### Basic Characteristics of the Sample (Table 5 – Unweighted Estimates)

**Table 5:** Basic Characteristics of sample (Unweighted Estimates) by gender

**Characteristic**	**Total**	**Males**	**Females**
**Marital Status**	*n(%)*	*n(%)*	*n(%)*
Single/Other	1084 (54.3)	394 (58.2)	690 (52.3)
Married/Cohabiting	912 (45.7)	283 (41.8)	629 (47.7)
**Highest Education Level**			
All Age or less	982 (49.3)	372 (37.9)	305 (30.2)
Secondary or above	1009 (50.7)	610 (62.1)	704 (69.8)
**Employed Full-Time**			
No	1174 (59.6)	343 (50.9)	831 (64.0)
Yes	797 (40.4)	330 (49.0)	467 (36.0)
**Respondent is employed**			
No	583 (29.6)	105 (15.6)	478 (36.8)
Yes	1389 (70.4)	568 (84.4)	821 (63.2)
Age-specific employment rates*			
24 yrs and under	259 (67.2)	117 (83.0)	142 (58.2)
25 – 34 yrs	350 (74.3)	128 (91.4)	222 (67.1)
35 – 44 yrs	316 (80.8)	120 (96.0)	196 (73.7)
45 – 54 yrs	194 (82.2)	81 (95.3)	113 (74.8)
55 – 64 yrs	150 (62.2)	67 (76.1)	83 (54.3)
65 yrs and older	111 (47.4)	52 (57.8)	59 (41.0)
**Subject has second occupation**			
No	1551 (79.8)	470 (70.7)	1081 (84.5)
Yes	393 (20.2)	195 (29.3)	198 (15.4)
**Reports religious affiliation**			
No	224 (11.1)	135 (19.9)	89 (6.7)
Yes	1788 (88.9)	545 (80.2)	1243 (93.3)
**Actively practises religion**			
No	793 (44.2)	308 (54.7)	485 (39.3)
Yes	1003 (55.9)	255 (45.3)	748 (60.7)
**Social Status^+^**			
Low	668 (33.2)	218 (32.1)	450 (33.8)
Middle	1120 (55.7)	391 (57.5)	729 (54.7)
High	224 (11.1)	71 (10.4)	153 (11.5)

* – This gives distribution of ages among those employed; ^+ ^– Interviewer's assessment

Approximately 46% of persons recruited into the study were either married or cohabiting in a common-law relationship but women were more likely to be in these types of union than men (47.7% vs 41.8%, p < 0.001). Just over 70% of subjects were employed with a greater frequency among men compared to women (84.4% vs 63.2%, p < 0.001) (data not shown) but full-time employment was reported by only 40.4% and was more frequent among men (49.0%) than women (36.0%, p < 0.001). Employment rates were highest in the 35–44 and 45–54 year olds, 80.8% and 82.2% respectively and lowest in the oldest age group, 65–74 y.o., at 47.4%. This pattern was present in both males and females.

Twenty per cent of the sample, and a larger proportion of males, [29.3%, compared with females, 15.4% (p < 0.001)], reported having a second occupation. Religious affiliation was reported more frequently among women (93.3%) compared to men (80.2%) (p < 0.001) and a larger proportion of women reported actively practising their religion (60.7% vs 45.3%, p < 0.001).

The gender-specific distributions of observer-perceived social class did not differ significantly and, overall, approximately 33% were perceived as lower class, 56% as middle class and 11% as upper class.

### Response Rates to Specific Questions (Table 6)

**Table 6:** Non-Response Rates (%) to Questions by Categories

Demographic Questions	Total	Males	Females	Rural Residents	Urban Residents
Level of Education	0.8	0.2	1.1	0.4	1.2
Employment status	1.4	0.6	1.9	1.4	1.5
Possession of second occupation	3.4	2.2	4.0	2.8	4.1
Religious affiliation	1.2	1.0	1.3	1.0	1.6
					
Family's Medical History	Total	Males	Females	Rural Residents	Urban Residents

Mother's history of hypertension	6.6	6.9	6.4	7.0	6.0
Father's history of hypertension	9.3	9.0	9.5	9.0	9.7
Mother's history of diabetes	5.3	5.3	5.3	5.5	5.0
Father's history of diabetes	7.1	7.1	7.1	7.1	7.0
					
Own Medical History	Total	Males	Females	Rural Residents	Urban Residents

Time of last BP measurement	1.6	2.4	1.2	1.0	2.3*
Told suffering hypertension	1.7	2.7	1.2*	1.6	1.8
Told suffering diabetes	1.4	1.3	1.4	0.9	2.0*
Told had heart attack	1.0	0.6	1.2	0.4	1.7**
Told suffered stroke	0.8	0.6	0.8	0.3	1.2*
Told suffering cancer	1.7	1.8	1.7	1.1	2.4*
					
Social Amenities	Total	Males	Females	Rural Residents	Urban Residents

Type of toilet facilities	0.6	0.2	0.8	0.1	1.1**
Household size (persons)	0.7	0.4	0.8	0.3	1.0*
No of rooms in house	1.0	1.0	1.0	0.6	1.5*
Income	30.3	27.9	31.5	28.8	32.0
					
Sexual Practices	Total	Males	Females	Rural Residents	Urban Residents

Sexually active in the past year	1.3	0.9	1.5	1.7	0.9
Ever had sexually transmitted disease	1.3	0.8	1.6	1.8	0.8*
Frequency of intercourse	9.4	6.7	10.8**	9.7	8.9
Usually uses condom	8.6	4.2	10.8**	8.6	8.6
Number of sex partners in previous year	23.1	15.8	26.8**	21.0	25.4*
Sexually active in the past year	1.3	0.9	1.5	1.7	0.9

Failure to respond to questions on demographic issues including education, employment status and religious affiliation was infrequent, occurring in less than 2% of cases, except in the enquiry about a second occupation where non-response ranged from 2% to 4%. Even at these low levels there were some statistically significant between-group differences with females having marginally higher non-response than males on questions on education and employment; and urban residents having higher non-response rates than rural counterparts on level of education achieved. Questions on women's health elicited high response rates with the highest non-response on questions about diabetes in pregnancy where 4% of women failed to provide a response. Questions enquiring about the health of subjects' parents resulted in non-response in 5–9% of cases with no differences on each question between genders or urban/rural residents. Non-response was consistently higher in both genders in questions asking about history of fathers' health compared to mothers' health. Non-response was lower on personal medical history compared to family history, remaining consistently below 3%. Some of these differences were statistically significant with urban residents having higher non-response rates than rural counterparts. Gender differences were infrequent but men were less likely to provide an analyzable response on the history of hypertension.

Table 6 also shows within and across gender and urban-rural groups, low non-response (< 2%) to items eliciting information on sexual activity or contraction of sexually transmitted infections. In comparison, questions on frequency of intercourse and condom usage resulted in non-response of 8–11% and women were significantly less likely to respond. Enquiry about the number of sexual partners in the last year resulted in the highest levels of non-response (15.8–26.8%) and compared with men, women provided significantly less analysable data on this issue as did urban residents compared with rural residents.

Non-response was low on questions of safety in motorized travel (wearing helmet and seat belts), history of injuries, witnessing violent acts and carrying offensive weapons (<2%) with occasional gender and urban rural differences (data not shown). Non-response was also low for questions on mood (maximum 3.9% non-response) but more variable for questions on physical activity. Less than 2% failed to describe physical activity at work but up to 8% failed to describe leisure time physical activity and frequency of physical activity (data not shown). A large percentage of respondents provided data on whether they smoked tobacco or drank alcohol (<2% non-response) but 8–10% did not provide analyzable responses to the quantity of cigarettes and alcohol and the age when smoking started (data not shown).

Social status questions were usually answered with the stark exception of income where approximately 30% of subjects failed to provide a response and this was consistent across genders and area of residence. This is by far the highest level of non-response among all questions and groups of questions. In other areas, except for some questions on sexual practices, non-response was consistently below 10% and very often less than 5%.

We examined whether persons requiring multiple visits (1, 2, or ≥3 visits) differed in their frequency of item non-response. The frequency of item non-response on income decreased with increasing number of visits required for recruitment (31.8%, 26.3% and 21.7%; p < .05 for trend). There was no trend in response to number of sexual partners in the last year.

Whereas 67.1% of non-responders to the question on income replied "don't know" only 3.1% of non-responders to question on number sexual partners gave the same response. For income non-response, the proportion of "don't know" among non-responders varied with age group at 78.0%, 68.3%, 65.2%, 58.0%, 61.3% and 74.% for the 15–24, 25–34, 35–44, 45–54, 55–64 and 65–74 age groups respectively.

### Correlates of non-response to questions on income (Table 7)

**Table 7:** Odds ratios with 95% confidence intervals (in brackets) and percentages of non-responders indicating the factors associated with non-response to questionnaire items eliciting information on income.

	**Withholding income data**			
**Sub-population**	**Weighted estimates**		Un-weighted estimates	

	**%**	**OR (95% C.I.)**	**%**	**OR (95% C.I.)**

Highest education level**				
**Primary & All Age**	**24.7**	1.0	27.3	1.0
**Secondary**	**34.7**	1.73 (1.24, 2.41)	33.1	1.53 (1.17, 1.99)
**Tertiary**	**27.7**	1.55 (0.82, 2.93)	27.7	1.43 (0.93, 2.18)
Unemployed**				
**Yes**	**42.4**	1.0	40.5	1.0
**No**	**25.1**	0.48 (0.34, 0.67)	24.6	0.52 (0.41, 0.66)
Age categories (years)**				
**15–24**	**43.5**	1.0	41.9	1.0
**25–34**	**21.1**	0.37 (0.26, 0.53)	22.2	0.42 (0.30, 0.58)
**35–44**	**26.2**	0.56 (0.36, 0.85)	26.6	0.58 (0.42, 0.81)
**45–54**	**21.5**	0.55 (0.35, 0.86)	22.7	0.58 (0.39, 0.87)
**55–64**	**31.3**	0.77 (0.47, 1.26)	32.3	0.79 (0.53, 1.18)
**65–75**	**38.4**	0.99 (0.64, 1.53)	39.9	1.07 (0.72, 1.60)
**Gender**				
**Male**	**27.9**	1.00	27.9	1.00
**Female**	**32.8**	0.98 (0.71, 1.35)	31.5	1.01 (0.81, 1.28)
Health Regions**				
**South East**	**25.8**	1.00	25.0	1.00
**North East**	**20.7**	1.07 (0.39, 2.96)	22.9	1.27 (0.88, 1.83)
**South**	**45.0**	3.15 (1.26, 7.90)	45.5	3.32 (2.45, 4.49)
**West**	**36.3**	2.26 (1.18, 4.35)	35.0	2.30 (1.68, 3.15)
**Socioeconomic Status^1^**				
**Low**	**30.1**	1.00	29.2	1.00
**Middle**	**30.6**	1.08 (0.74, 1.59)	31.2	1.12 (0.89, 1.42)
**High**	**29.8**	0.95 (0.43, 2.10)	29.5	1.03 (0.65, 1.61)
**Urban resident**				
**No**	**28.6**	1.0	28.8	1.0
**Yes**	**32.3**	1.54 (0.77, 3.07)	32.0	1.66 (1.29, 2.15)

** – P < 0.01

^1 ^These categories represent socioeconomic status as perceived by the interviewer

Further analysis of the response to questions about income showed that there were significant differences between educational levels with persons achieving secondary education having a 34.7% non-response rate compared to 24.7% and 27.7% for those with primary or lower and tertiary levels respectively. The unemployed (42.4%) were less responsive than the employed (25.1%) and youngsters <25 years old (44%) and mature persons 55 years and older (36.1%) provided less data compared to those in the intervening years of age (21.5–26.2%). There is little difference between weighted and unweighted rates of the non-response to questions on income. There were no differences between genders, areas of residence or observer-perceived social status categories but the health regions showed significant differences ranging from a low non-response of 20.7% in the NERHA compared to a high of 45.0% in the SRHA.

In weighted multiple logistic regression analyses accounting for all tabulated variables (Table 7), significant independent associations were detected with educational level, employment status, health region and age.

## Discussion

### Item Non-response

In this national survey enquiring into a wide range of health issues, using an interviewer administered questionnaire we have demonstrated that free-living participants demonstrate a wide range of responsiveness to the questions posed to them. The non-response rate (30%) was highest for questions on household income both within and across genders and area of residence (urban/rural). The importance of socioeconomic status (SES) to health and the frequent use of income data to assign SES makes it important to explore whether income data may be seriously affected by non-response bias [20-22]. Compared to persons 24 years or younger, all the older age groups were significantly less likely to withhold income information. The relationship with age was not linear but U-shaped, with the youngest (<25 years) and the oldest (65–74 years) showing the highest non-response, 43.5% and 38.4% respectively, to income questions. There are few similar data on income non-response in health surveys from developing countries but in a previous study from Jamaica 18% of respondents did not report on their income; however in that survey the correlates of non-response were not reported [23]. The findings in this study contrast in extent and trend with that estimated in a developed country as reported by Turrell [13] who found a lower (9.8%) frequency of income non-response in Australia and a near linear trend of increasing non-response with age compared to the U-shaped pattern seen in our study. A similar pattern in item non-response to that described by Turrell was found among an elderly cohort in the USA [24]. The higher income non-response among the young in our study could result from young persons being less trusting than their more mature counterparts or may be driven by feelings of embarrassment because they have less information to report. We note that the proportion of "don't know" among income non-responders is high in all age groups (lowest 58% in the 45–54 year olds). Ignorance of household income as against personal income may be the main explanation but reluctance to reveal income cannot be excluded. Inclusion of a questionnaire item labelled "no income" may be one way to surmount high non-response as persons who do not generate income may be encouraged to select this item if it reflects their experience. It is uncertain what impact this would have on our survey as we enquired about household rather than personal income. It is expected that this information may be unequally available in different members of the household depending on their various roles.

This relationship with age remains independent of employment status in multivariate analysis but it is to be noted that the lowest non-response rates are in the age groups with the highest levels of employment (25–54 years old) and we cannot entirely rule out employment status as an explanation for the age trend. Other factors which showed independent statistically significant relationship with income non-response include educational level, regional health authority and employment status. Persons having secondary level education were more likely (OR = 1.73, 95% CI = 1.24 to 2.41) to withhold income data compared with persons educated to the primary level only. Persons in the Southern and Western regional health authorities were also more likely to withhold income data compared with persons in the South-eastern region. We showed no relationship between income-non-response and gender, urban versus rural dwelling and interviewer-perceived socioeconomic status (SES). These findings are different from the Australian data [13] where there was no relationship to educational level; a strong relationship with SES; and less reluctance among the unemployed to report income data, 4.1% vs 10.9%, respectively, in the Australian data, compared to 42.4% vs 25.1% in our study. Turrell [13] also makes reference to eight (8) comparable studies using face-to-face interviews and where income non-response ranged from 10% to 25% but was less than 20% in six of these eight studies. All these studies were carried out in developed countries of Europe and North America. These data suggest that non-response to questions on income may be more frequent in developing compared to developed countries and demonstrate a systematically different pattern with respect to employment status.

The statistically significant differences suggest an association between these characteristics and participants' non-response to particular types of questions, a phenomenon that can seriously distort estimates based on such results[25]. It may be necessary to augment future analysis of income data with imputation of missing values. Results of data analysis with and without missing data need to be compared to determine whether the results differ markedly. Inference should be based on the data set that better represents the population.

It has been shown in previous studies [13] that income non-response may be lower in mail surveys than in face-to-face interviews. On the other hand non-response is less where income category rather than exact income is requested. Thus the Jamaican Health and Lifestyle survey was at an advantage on the latter condition and by current standards would have had a good chance of eliciting a high response rate. These data are important as the established and currently topical relationship between SES and health requires that reliable and valid data be available on which to classify SES groups[22,26,27]. It would appear that reported income data may not be an entirely reliable and valid estimate of social class and that its validity may differ across age groups, employment status and educational levels.

In the context of our social norms it may have been speculated that free-living individuals would be less willing to report on sexual activity than on income but non-response frequencies were much less on sexual activity than on income in this study. The highest non-response frequency in respect of sexual activity was in regard to number of sexual partners (23.1%). In contrast to income non-response, there were gender differences (Table 6) where females provided significantly less analysable data on three aspects of sexual activity. These gender differences in responding to sensitive sexual issues have been demonstrated in other studies in both telephone interviews and in telephone interview with computerized questionnaire method. In one study telephone interview methods appear to elicit higher response rates of greater than 90% in both males and females [14].

Non-response was consistently higher in both genders in questions asking about history of fathers' health (up to 10%) compared to mothers' health. This probably is a reflection of the higher level of paternal absenteeism in the Jamaican society such that children tend to know less about the health of their fathers and this is in keeping with the fact that over 80% of children in Jamaica are born to unmarried women [4]. Where family history may be an important marker of risk, for example in cardiovascular diseases and cancer, the assessment of this risk may be compromised by non-response or ignorance of respondents. Further study would be required to assess factors that influence the child's awareness of illnesses suffered by their parents.

### Comparison with national data

Representativeness of the sample studied in this survey may be assessed by comparison of its findings with national data available from other sources. In this study important socio-demographic estimates include a 75.8% (male 87.3%; female 64.4%) employment rate and this compares to a reported 87% employment rate (male 90.3%; female 82.4%) in the Economic and Social Survey of the Jamaica [28]. Our report is likely to be an underestimate as it includes persons who may not be in the labour force market as defined by the Planning Institute of Jamaica [28]. We also compared our estimates of union status with the output from the 2001 census. Overall, 40.9% of our sample was in a legal marriage or common-law cohabitation (male 36.8%; females 44.9%). This compares with 40.2% (male 41.4%; female 39.0%) obtained from the 2001 census data. These comparisons strengthen our confidence in the representativeness of the sample that was studied. In this survey we demonstrated that large field teams can be trained to produce reliable results in national surveys.

### Response Rates

Overcoming non-response can be a costly exercise. According to Politz and Simmons [29] the cost of each succeeding visit is higher than that of the previous one. Within and across health regions, more than 20% of the subjects required at least one call-back. As a consequence of the increased cost per unit of information derived from call-backs, Hansen and Hurwitz (1946) as cited by Politz and Simmons [29] recommended revisiting a sub-sample rather than the entire group of the "not at homes". These authors concede, however, that bias and increased sampling error can be a consequence of such sub-sampling. Another strategy recommended by these authors is the weighting of the responses based on the probability of subjects being at home where such information was available from prior studies.

Failure to obtain measurements on participants in a chosen sample introduces error [10,30]. This can result from failure to locate individuals or their refusal to answer questions when located. In our study the person eligible to be interviewed was selected at random and was not replaced by another if the selected person declined. Efforts to reduce non-response due to "not-at-homes" are challenged by this random selection. In order to minimise error, the study adopted the policy of allowing a minimum of 3 call-back visits if the selected household member was not at home at the first visit.

Other factors which affected response rates in our survey include difficult terrain, unfavourable weather conditions and tension and insecurity resulting from violence in certain urban communities. In our study, these problems were addressed by the use of special vehicles and the establishment of liaison with influential community members. This approach was critical to gaining community support for and participation in the survey. Recruitment was also facilitated by the extension of outreach health services to non-eligible persons in order to build goodwill. While these actions resulted in improved response rates, it is difficult to assess whether they increased bias in the estimates obtained.

### Sample Selection

The study guidelines for selecting a single individual from among the eligible persons in a given household used a method developed by Kish [18]. The strategy of selecting a single person was useful for this study as one person might not necessarily be able to report accurately for another person. In addition, as intra-household correlation might be very high, multiple selections per household could not be encouraged. Thus, there was increased independence of the responses between selected subjects. It is recognized that this independence or the lack of it, within a given household will depend on the variable being measured and selecting a single subject per household may sometimes be unnecessary. Nevertheless we chose this approach in order to err on the side of caution. The method also reduces selection bias by giving equal probability of selection to all eligible members of the household and preventing replacement of the selected person within a given household thereby improving the representative nature of the sample. Some authors have recognized however that for larger households the probability of selection is not equal for all persons [31]. This may account for the discrepancies between our sample and the source population when distributions of age and gender are compared. Information on household size would allow for further exploration of this phenomenon – unequal probability of selection – but these data are not available from our study.

In an effort to lessen bias in the estimates of population parameters, a consequence of non-response and sampling frame under coverage [32], post-stratification weights were applied to these data. As a consequence of this measure, the distributions of counts for 5-year age by sex categories within the study sample patterned the distribution in the Jamaican population. Use of the technique is very relevant to a study such as ours in which we wish to make inference about the Jamaican population based on our study sample. Post-stratification also lessens the variability of estimators obtained using these weights [32] and, consequently, we expect enhanced validity of the inference based on the collected data. Our analysis also accounted for the sample design (multistage cluster design) and incorporated post-stratification weights thereby allowing for more conservative estimates of and greater confidence in demonstrated associations [33].

The excess of women in our sample has been a feature of surveys in the region and elsewhere [15,34,35]. It has been suggested that in the case of Jamaica, males are less likely than females to meet the place of residence criterion of sleeping at least three nights per week at the address [35].

This is the largest national health survey to be done in Jamaica and encompasses a larger age range (15–74 years) than the previous survey in 1993 which reported on persons 15–49 years old [36]. We recruited a national sample of the Jamaican population selected by multistage sampling and as in previous studies in Jamaica and elsewhere it proved easier to recruit females than males to this survey [17,18]. This survey, comparable in size and scope with similar studies worldwide, [37] will provide useful information on disease burden and risk factors for leading causes of morbidity and mortality in the Jamaican population but of equal importance it presents an opportunity to examine the logistics, costs and relative biases associated with doing studies of this nature in other populations at a similar stage of development.

## Conclusion

In the study we have found that recruiting male subjects is more challenging than females. This is in keeping with previous experience in Jamaica and other countries. There is a small age related variation in the efficiency of carrying out surveys as estimated by per-person time to complete interviews with older persons requiring a longer time; this may be important in large surveys. Survey response rates were good, ranging from 81–98% but regional differences were statistically significant. The highest level of item non-response was on questions related to income and sexual activity. Non-response to questions on income was significantly related to educational level, employment status, health region and age. The extent and pattern of item non-response to income is different from developed countries being higher and varying differently with age. Responders who report "don't know" may be genuinely different from others who fail to provide a response but this may depend on the nature of the question.

Developing countries may have different types of bias in health surveys when compared to developed countries.

## Competing interests

The author(s) declare that they have no competing interests.

## Authors' contributions

RW was principal investigator and along with EW, PF and DA were responsible for the proposal development and obtaining grant funds.

JM was the project coordinator responsible for field coordination and data collection.

NY, NZ and CW were responsible for data management and data analysis.

RW, NY, NZ, PF, MT-R and TFe were responsible for data interpretation.

RW and NY drafted the manuscript.

All authors reviewed the manuscript, participated in its revision and were responsible for important intellectual content.

## Pre-publication history

The pre-publication history for this paper can be accessed here:


